# Supplementary material for: The Severity of Pain in Prostate Biopsy Depends on the Biopsy Sector
Source: J Pers Med. 2023 Feb 27;13(3):431. doi: 10.3390/jpm13030431 (PMC10057203; doi:10.3390/jpm13030431)

BIOPSY UNDER CONTROL OF MRI AND TRUS FUSION - PATIENT CARD

DATE OF BIOPSY:…………………………………………….

NAME AND SURNAME:……………………………………………………………………………………………………..

A. BODY WEIGHT:………………HIGHT:…………………………..PHONE NO:………………………………………

C) NICOTINISM: **YES/NO** PACK-YEAR…………………………

D) MEDICAL INTERVIEW ON PROSTATE CANCER IN THE FAMILY: **YES/NO**

E) DRE: **CORRECT/INCORRECT**

F) BIOPSY ON MEDICAL INTERVIEW: **YES/NO** BIOPSY NUMBER…………

IF YES --- HISTOLOGICAL - PATHOLOGICAL TEST RESULTS …………………

G) PSA VALUE:……………. FPSA:………. THE HIGHEST PSA WALUE IN INTERVIEW:………………

H) INFLAMMATION OF THE PROSTATE IN INTERVIEW: **YES/NO**

I) DISTURBANCES OF MICTION: **YES/NO** NYCTURIA: **YES/NO**

J) STATE AFTER TURP: **YES/NO** USE OF TESTOSTERONE: **YES/NO**

K) PHARMACOLOGICAL THERAPY BPH: **YES/NO** NAME OF THE DRUG………………………………

L) HISTORY OF PELTER OPERATIONS **YES/NO** M) RADIOTERAPHY OF PELVIS **YES/NO**

N) CHRONIC DISEASES…………………..………………………………………………………………………….

O) BIOPSY TIME (MIN)…………………………… P) COMPLICATIONS: **YES/NO** ………………………….

R) TRUS: WxHxL……………………………. vol…..... TRUS TNM:……………………………………………..

S.) MR: WxHxL……………………………… vol………. MR TNM:………………………………………………

PIRADS: …………………………………………………….

ROI NUMBER:…………………………………………….

ROI DIMENSIONS: …………………………………….

IS ROI SUSPECT IN TRUS? **YES/NO**

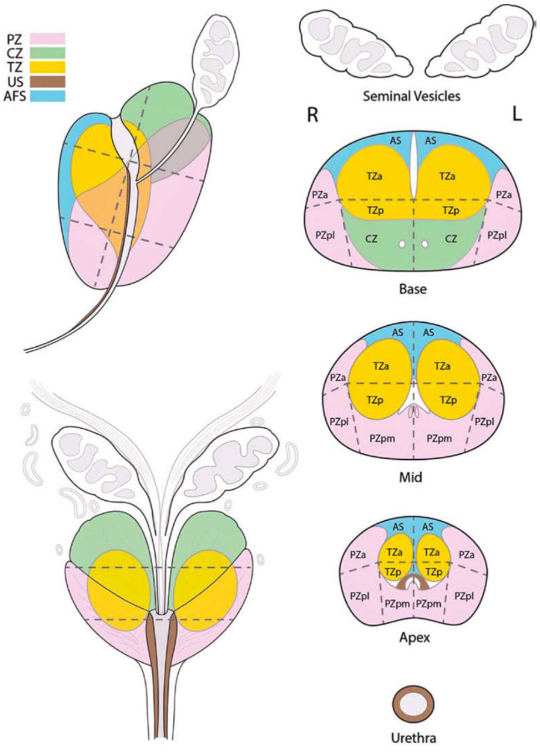

Supplement: Supplementary file 1 [file jpm-13-00431-s001.zip › jpm-2174657-supplementary/Supplementary Data S1.pdf]
