# Supplementary material for: The Severity of Pain in Prostate Biopsy Depends on the Biopsy Sector
Source: J Pers Med. 2023 Feb 27;13(3):431. doi: 10.3390/jpm13030431 (PMC10057203; doi:10.3390/jpm13030431)
Supplement: Supplementary file 1 [file jpm-13-00431-s001.zip › jpm-2174657-supplementary/Supplementary Data S2.pdf]

Patient survey

Patient data:……
………
………

A. Answer the questions below using the pain scale.

0	1	2	3	4	5	6	7	8	9	10
No pain		Mild		Moderate			Strong			Unbearable pain

1. How would you rate the pain throughout the procedure?………………………………………………0 1 2 3 4 5 6 7 8 9 10
2. How would you rate the pain during digital rectal examination?…………………………………………0 1 2 3 4 5 6 7 8 9 10
3. How will you rate the pain while inserting the ultrasound probe into the rectum?……………0 1 2 3 4 5 6 7 8 9 10
4. How would you rate the pain during the administration of anesthesia? ……………………………0 1 2 3 4 5 6 7 8 9 10
5. How would you rate the pain during maneuvering?……………………………………………………………0 1 2 3 4 5 6 7 8 9 10
6. How would you rate the pain while taking the samples? …………………………………………………0 1 2 3 4 5 6 7 8 9 10

B. How anxious or tense were you at the time of the biopsy? (scale of anxiety)

0…..1…..2…..3…..4…..5…..6…..7…..8…..9…..10
No anxiety Unbearable anxiety

C. What discomfort did you feel during the biopsy? (scale of discomfort)

0…..1…..2…..3…..4…..5…..6…..7…..8…..9…..10
No discomfort Severe discomfort

D. If there was a medical need for a repeat biopsy, how much would you be inclined to undergo a repeat biopsy? (tolerance scale)

0…..1…..2…..3…..4…..5…..6…..7…..8…..9…..10
Very willingly Never
