# Supplementary figures and images for: The Severity of Pain in Prostate Biopsy Depends on the Biopsy Sector
Source: J Pers Med. 2023 Feb 27;13(3):431. doi: 10.3390/jpm13030431 (PMC10057203; doi:10.3390/jpm13030431)

PZ  
 CZ  
 TZ  
 US  
 AFS

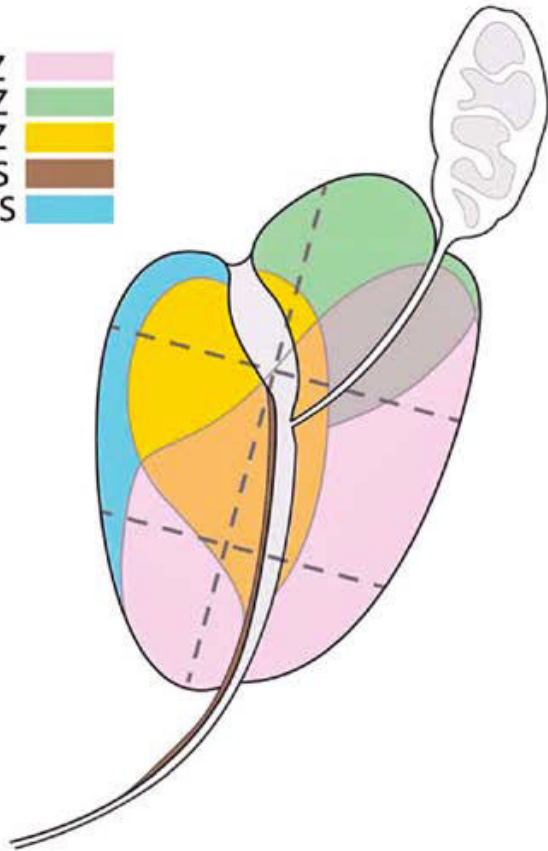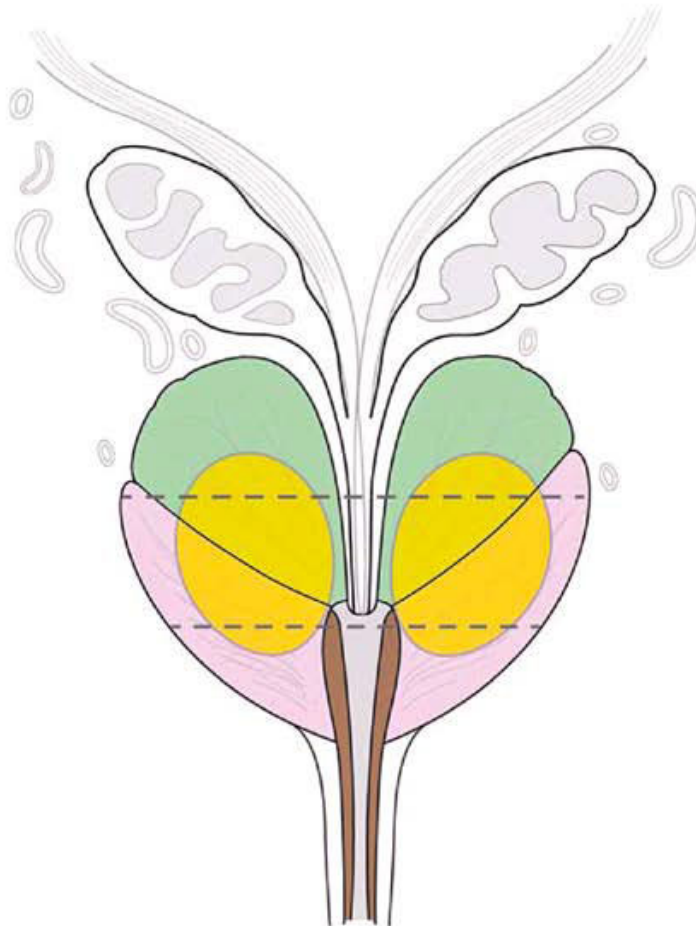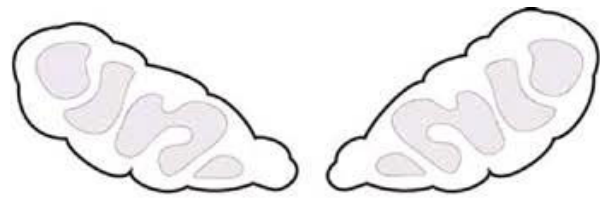

Seminal Vesicles

R

L

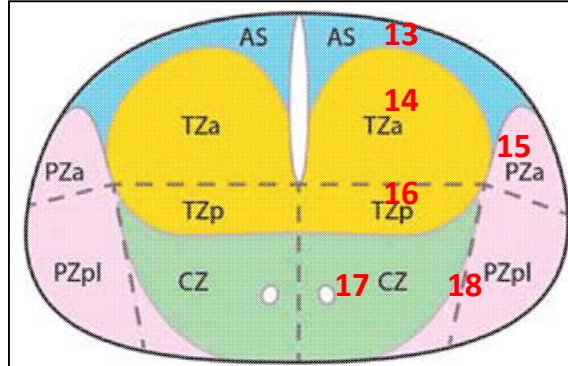

Base

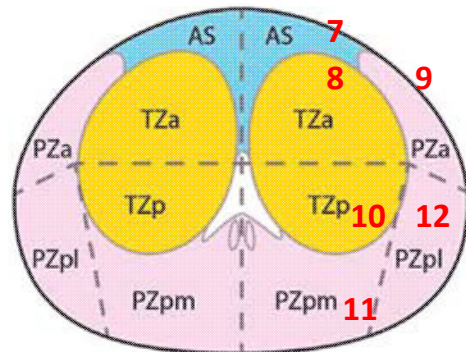

Mid

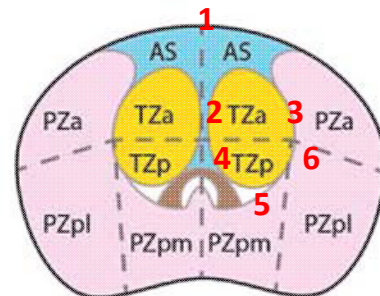

Apex

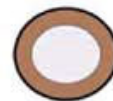

Urethra

Supplement: Supplementary file 1 [file jpm-13-00431-s001.zip › jpm-2174657-supplementary/Supplementary Data S3.pdf]
